# Comprehensive Pilot Analysis of Oral Hypofunction Among Swiss Adults

**DOI:** 10.1155/ijod/8852345

**Published:** 2025-04-10

**Authors:** Murali Srinivasan, Lisa Takeshita, Cláudio Rodrigues Leles, Dusit Nantanapiboon, Manabu Kanazawa, Koichiro Matsuo

**Affiliations:** ^1^Clinic of General, Special Care and Geriatric Dentistry, Center for Dental Medicine, University of Zürich, Zurich, Switzerland; ^2^Faculty of Dentistry, Federal University of Goiàs, Goiania, Brazil; ^3^Dental Material Research and Development Center, Faculty of Dentistry, Chulalongkorn University, Bangkok, Thailand; ^4^Department of Operative Dentistry, Faculty of Dentistry, Chulalongkorn University, Bangkok, Thailand; ^5^Department of Gerodontology and Oral Rehabilitation, Graduate School of Medical and Dental Sciences, Tokyo Medical and Dental University, Tokyo, Japan; ^6^Department of Oral Health Sciences for Community Welfare, Graduate School of Medical and Dental Sciences, Tokyo Medical and Dental University, Tokyo, Japan

**Keywords:** geriatric dentsitry, masticatory function, occlusal force, oral diadochokinesis, oral hypofunction, swallowing function, tongue pressure

## Abstract

**Objectives:** This study aimed to explore the effects of age and sex on oral function, specifically focusing on the prevalence of oral hypofunction (OHF) in different age groups.

**Materials and Methods:** A total of 126 healthy adults, evenly distributed across six age groups (20–29, 30–39, 40–49, 50–59, 60–69, and 70+ years), participated, with an equal number of men and women in each group. OHF was assessed using seven key oral function parameters: oral hygiene, oral dryness, occlusal force, oral diadochokinesis (ODK), tongue pressure, masticatory function, and swallowing function. Body mass index (BMI) and hand grip strength (HGS) were also measured. Data analysis included descriptive statistics, bivariate comparisons with effect size(ES) calculation, and exploratory factor analysis to identify underlying domains affecting oral function (*p* < 0.05).

**Results:** Older age groups exhibited a marked decline in occlusal force (*p* < 0.001), ODK (*p* < 0.001), tongue pressure (*p*=0.002), and masticatory function (*p* < 0.001), with moderate to large ESs. Factor analysis revealed three domains: chewing-related, tongue-related, and oral status. The chewing-related and tongue-related domains were impacted by age (*p* < 0.001), while the oral status domain showed no significant age-related changes (*p*=0.135). Additionally, the prevalence of OHF increased with age, with older participants showing a higher number of failed oral function tests. No significant differences in oral function were observed between sexes.

**Conclusion:** The findings suggest an influence of age in oral function, particularly in chewing and tongue-related activities, contributing to increased OHF in older adults. These results highlight the importance of early detection and targeted interventions to maintain oral health and function in the aging population, with a focus on preserving chewing and tongue-related capabilities. The oral status domain, however, did not correlate with age, suggesting other factors might be involved in its deterioration.

## 1. Introduction

Oral function is integral to overall health and well-being [1–3]. Essential activities such as eating, speaking, and swallowing depend on the coordinated efforts of the mouth, teeth, tongue, and associated structures. Effective mastication and swallowing are critical components of the digestive process, ensuring that food is adequately broken down and nutrients are absorbed efficiently [4, 5]. As such, any decline in oral function can have significant repercussions on nutritional intake and, consequently, on general health [6, 7]. When oral function deteriorates, individuals may begin to avoid hard-textured foods and opt for softer diets [8, 9]. This change in dietary habits can lead to a suboptimal nutritional state, which is a primary contributor to malnutrition [10]. Malnutrition is not just a concern of weight loss or lack of caloric intake but is a significant risk factor for physical frailty [11]. Physical frailty is characterized by decreased strength, endurance, and physiological function, which increases vulnerability to health deterioration and has been shown to be linked to oral frailty [11, 12]. Moreover, poor nutrition can impair immune function, delay wound healing, and reduce the overall quality of life. Beyond the physiological impacts, oral health significantly affects social interactions and psychological well-being. Individuals with compromised oral function may feel self-conscious about eating in public or engaging in social activities involving food [8]. This self-imposed social isolation can lead to feelings of loneliness and depression, further exacerbating the overall decline in health [8]. Therefore, the social dimension of oral health is crucial, as it contributes to both mental and physical well-being.

Oral hypofunction (OHF) is a term introduced by the Japanese Society of Gerodontology to describe the deterioration of oral functions, particularly in older adults [13]. OHF is not a singular condition but a composite of various sub-symptoms, each contributing to the overall decline in oral capability. The seven subsymptoms of OHF include decreased tongue pressure, diminished occlusal force, impaired ODK, reduced saliva secretion, altered swallowing function, and changes in oral hygiene and oral dryness. A diagnosis of OHF is made when at least three of these symptoms meet the established cutoff criteria [13]. Given the complexity of oral functions and the potential for one function to compensate for another, it is vital to quantitatively assess the decline in specific oral capabilities. Such assessments enable healthcare providers to monitor the oral condition effectively and intervene promptly. For instance, measuring tongue pressure, occlusal force, and ODK can provide valuable insights into a patient's oral health status. These metrics help identify early signs of deterioration, allowing for timely therapeutic interventions to prevent further decline.

Physical strength, often measured by hand grip strength (HGS) and skeletal muscle mass, is known to decline with age [14, 15]. These variables are routinely used as indicators for conditions like sarcopenia. Despite abundant normative data on physical strength and body composition, there is a paucity of comprehensive studies on oral function across the adult lifespan in the Swiss population. Some research has evaluated specific aspects, such as tongue pressure, in different countries, but a holistic examination of OHF categories across all age groups remains limited. This gap in knowledge underscores the need for studies that explore the relationship between oral and physical functions across diverse populations.

This study hypothesizes that oral function correlates with physical function regardless of age or sex and that it diminishes with age. To test this hypothesis, the study aims to investigate age and sex differences in oral function across various age categories, following the established OHF criteria. The study sought to provide a comprehensive understanding of how oral function deteriorates with age and how this decline correlates with overall physical health.

## 2. Material and Methods

### 2.1. Study Design, Ethics

The study summary was sent to the relevant ethical committee (Kantonale Ethikkommission Zurich) and they decided that this study did not require a formal ethics approval (KEK-Zurich: BASEC-Nr.: Reg-2023-01106). The study was conducted in the Clinic for General-, Special Care-, and Geriatric Dentistry, in the Center for Dental Medicine, University of Zurich. The study was designed as an exploratory survey to prevalence of OHF in the local population living around the city of Zurich. This pilot study was planned to provide initial data for a future larger study with more outcomes. The study was designed as a single-center cross-sectional pilot survey.

### 2.2. Participants, Sample Size

Healthy asymptomatic adults were recruited across various age groups. A convenient sample of 20 per group was decided using the Pathfinder sampling method and equal number of men and women were included in each age group. The age groups were divided into the following age segments: 20–29 years, 30–39 years, 40–49 years, 51–59 years, 61–69 years, 70 years, and above groups. Participants were included if they were over 20 years of age without any neurological disorders and severe dysphagia. All participants provided a written informed consent prior to enrollment in this investigation. Participation was voluntary and no financial remuneration was given.

### 2.3. Outcome Measures/Endpoints

OHF was defined by the Japanese Society of Gerodontology (2016) as a combined decline of multiple oral functions. Seven elements of oral function along with their threshold limit for hypofunction are defined:

1. Oral hygiene: The oral hygiene was assessed using a bacteria detection device (Bacteria counter; Panasonic Healthcare, Tokyo, Japan) [16]. The threshold limit for the bacterial count was 10^6.5^ CFU/mL, hypofunction was for measures >10^6.5^ CFU/mL.
2. Oral dryness: A moisture checker sensor (Mucus; Life Co., Ltd., Saitama, Japan) was used to measure the moisture level of the buccal mucosa [17]. The dryness value as measured by the device must not fall below 27.0 for normal subjects.
3. Occlusal force: Maximum occlusal force was measured using a pressure-indicating film (Dental Prescale II; GC Corp., Tokyo, Japan), that provided the occlusal force distribution as well as the maximum occlusal force [18]. The normal threshold limit for the measured occlusal force was 200 N.
4. ODK: Participants were requested to rapidly repeat the syllables ‘pa', ‘ta', and ‘ka' for 5 s to measure ODK [19]. The number of syllables uttered was counted using a purpose-built smartphone app (SUNSTAR PATAKARA, version 1.1.0, 2021 Sunstar Inc.). The maximum count per second for each syllable was calculated and used for analysis. Normal threshold set was 6 iterations per second.
5. Tongue pressure: The maximum tongue pressure was measured with tongue pressure sensor balloon connected to a digital meter (JMS tongue pressure measuring instrument TPM-01; JMS Co. Ltd., Hiroshima, Japan) [20]. The device measured the pressure in kPa and the normal limit for this parameter was 30 kPa.
6. Masticatory function: Masticatory function was measured using a Gluco-Sensor (GS-II, GC Corp., Tokyo, Japan) [21]. The threshold to pass the masticatory function test was to achieve 100 mg/dL and more.
7. Swallowing function (EAT-10): The swallowing function was assessed using a self-administered questionnaire (10-item Eating Assessment Tool; EAT-10) [22]. Threshold score should remain below 3 for normal subjects.

Failure to achieve the predefined threshold limits would be considered hypofunction. A diagnosis of OHF was confirmed if the individual failed in three or more of these criteria.

Two physical parameters were also measured along with the parameters for OHF:

1. Body mass index (BMI): This was calculated from measured height and weight.
2. HGS: HGS was measured a digital hand dynamometer (Jamar Smart Hand Dynamometer, Lafayette Instruments, Lafayette, IN, USA). The highest value for each hand was used for analysis.

### 2.4. Data Analysis

Data analysis included descriptive statistics and bivariate comparison tests, according to the distribution patterns of data. The strength of association in nonparametric data was calculated as the Kruskal–Wallis H effect size (ES) − ES (*η*^2^ = *H*/*n*−1). The parameters for interpretation of the ES value were: 0.01–<0.06 (small effect), 0.06–<0.14 (moderate effect), and ≥0.14 (large effect). For calculation of the ES for a chi-square test with a linear trend, the Cramer's V measure was used, representing the magnitude of the association between categorical variables in a linear trend analysis (*V* = √ (*χ*^2^ / (*n*⁣^*∗*^*df*). The interpretation of Cramer's V was: small effect—less than 0.1, medium effect—between 0.1 and 0.3, and large effect—greater than 0.5.

Exploratory factor analysis was used to identify underlying variables, or factors, that explained the pattern of correlations within a set of observed variables represented by the seven oral function variables. The principal component analysis (PCA) as an extraction method, was used to explain the variance within the variables. A scree plot of the variance that was associated with each factor was displayed to determine how many factors should be kept, based on the distinct break between the steep slope of the large factors and the gradual trailing of the other factors. Factor loadings were considered significant when eigenvalues were >1, and varimax rotation with Kaiser normalization (rotation method) was used to minimize the number of variables that had high loadings on each factor. Statistical significance was set at *p* <0.05 for statistical inferences and the IBM-SPSS 24.0 software was used for all data analyses.

## 3. Results

A total of 126 participants were included in this study, 64 females (50.8%). The summary of data on the clinical and oral-related variables is detailed in Table 1. There was a significant effect of the age groups on the prevalence of obesity (BMI ≥ 25), use of dentures, and number of teeth present (*p* < 0.05). Likewise, the handgrip force was also reduced as the age group increases (*p* < 0.001).

Table 2 shows the variation in the mean values of the oral function parameters according to the age groups. Significant differences were found for the occlusal force (*p* < 0.001), ODK (*p* < 0.001), tongue pressure (*p*=0.002), and masticatory function (*p* < 0.001). Overall, these oral function parameters progressively deteriorate as the age groups are older. When the oral function parameters were expressed as the number of failed tests, significant (*p* < 0.001) higher numbers were observed in older age groups. Additionally, the magnitude of the effects of age on the function parameters were included in Table 2. All variables showed moderate (ES < 0.06) to large (ES < 0.20), which suggests that there are meaningful links between the age of the individuals and the quality of oral function measures.

**Figure 1:**
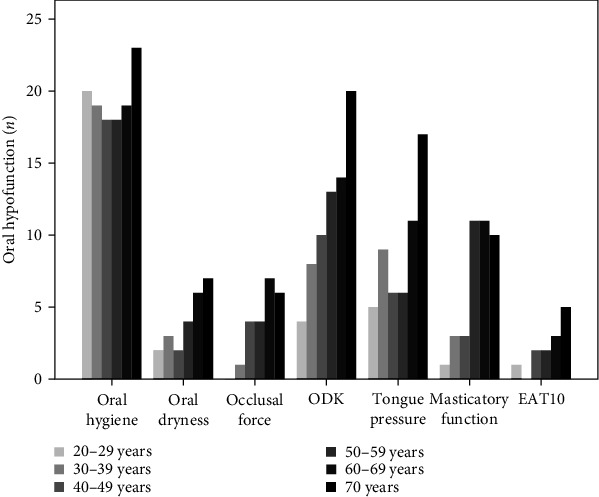
Frequency distribution (*n*) of participants with oral hypofunction, according to the age group and oral function parameter.

**Figure 2:**
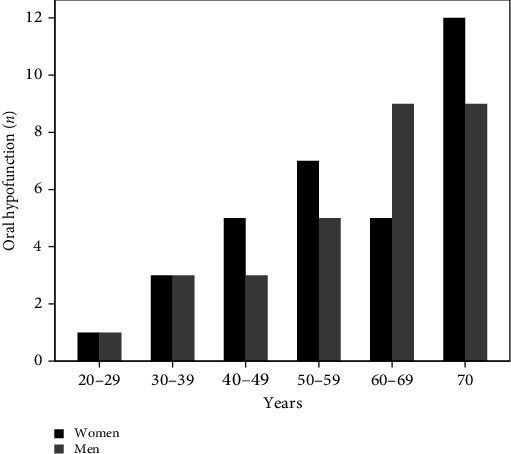
Frequency distribution (*n*) of participants with oral hypofunction, according to the age group and sex.

According to the cutoff values for definition of OHF for each oral function parameter, Figure 1 shows frequency distribution of participants with OHF, according to the age group for each oral function parameter. Chi-square for trend tests showed a significant association for occlusal force (*p*=0.002), ODK (*p* < 0.001), tongue pressure (*p*=0.002), masticatory function (*p* < 0.001), and EAT-10 (*p*=0.021). ES values showed a large effect for all the function parameters (ES > 5.6). Conversely, no difference was found in the prevalence of OHF according to sex (*p*=0.722) for the entire sample and by age group (*p* > 0.05), as shown in Figure 2.

Then, factor analysis was used to analyze the dimensionality of oral function parameters by reducing the set of oral function variables by extracting all their commonalities into a smaller number of factors (or domains).  Table 3 presents eigenvalues and respective percent of variance according to unrotated and varimax rotation methods. A three-factor model was obtained with a total variance explanation of 56.6%. An even variance distribution among the three significant factors was achieved and thus was chosen for further analysis. Subsequently, Table 4 lists the oral function variables sorted by size in descending order by their rotated loading coefficients on the factor for which they loaded the highest. The interpretation of the components identified the following factors: (1) chewing-related factor, (2) tongue-related factor, and (3) oral status. The first factor combined functions associated with jaw stroke movement to produce movement and chewing function, thus named as "chewing-related" domain. The second factor mainly addressed the issues of tongue and oropharyngeal function related to swallowing and speech, which was labeled as "tongue-related" domain. Lastly, the third factor comprised functions related to oral biology and hygiene control related to "oral status."

**Figure 3:**
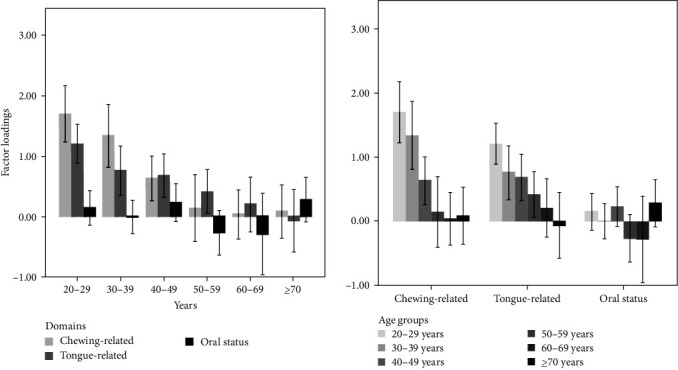
Factor loading values for the three domains, according to the age group. Data are expressed as means and 95% confidence intervals.

The summary values of the loading factors for each domain are presented in Figure 3, where significant effects of age were observed for both the chewing-related and tongue-related factors (*p* < 0.001), but not for the oral status domain (*p*=0.135). Therefore, findings suggest that the oral status domain, which includes the oral hygiene and oral dryness parameters were not useful for the identification of OHF.

## 4. Discussion

Overall, findings from this pilot cross-sectional study suggested that most of the oral function parameters were suitable to identify the changes due to age, and the significant decrease in oral function occurred progressively with the advancing in ages from 50–59years and 60–69years age groups. Moreover, the oral function variables related to chewing and tongue function were able to discriminate the occurrence of OHF.

Commercially available assessment tools for OHF have been tested in a previous study with healthy volunteers and showed good reliability [14, 23]. In addition, the oral function parameters used in this study are according to the Japanese Society of Gerodontology—JSG [13], that considered these seven parameters as key points within the conceptual model of OHF. However, the JSG recommended additional studies, especially from clinical studies and trials, to clarify and assess the validity and reliability of the proposed diagnostic criteria and shaping management strategies for older and frail subjects [13].

The relationship between reduced oral function or OHF and frailty in older subjects had been demonstrated in a large sample of community-dwelling adults aged 65–85 years in Japan, showing a near 60% of the older adults with OHF [24]. Functional parameters such as occlusal force, tongue-lip motor function, and swallowing function were significantly associated with the frailty status and OHF. However, they suggested that using of a single may be less reliable than a combination of oral function items which may increase the likelihood of identification of higher functional effects. Therefore, it was recommended that further research should focus on examining function items and combinations more closely associated with frailty [24].

Although our study was focused on healthy volunteers, we selected the age groups as a key factor for measuring the age-related changes in oral function, to validate the ability of the instruments to detect changes in the parameters as the age groups increase [25]. However, this is a limitation of the study design and sampling, and the inferences from this should be considered with caution since the measurement of OHF indicators should focus on subjects with impaired oral function due to poor dentition or motor disabilities caused by aging or pathophysiological disorders, to whom more crucial factors related to oral functional disorders might be identified [26]. Furthermore, long-term cohort studies would provide more robust evidence compared to the cross-sectional design of our study, since the incidence, causes, and prognosis of OHF could not be addressed in this study. Consequently, it was not possible to conclude whether the age factor is a causal link based on the strength of association, specificity, temporal relation, dose–response type relation, and among others [27]. Likewise, the inclusion of other confounding factors as independent variables in robust disease models, such as the dental status, comorbidities, and cognitive declining due to aging may be addressed in further research.

Finally, this study corroborates the evidence that the risk of OHF and frailty, may be increased with advancing age, and supports the recommendation that a reliable evaluation of oral function in transition ages and when early signs of worsening systemic health are detected [24]. Hence, the identification of poor oral function in adults in early stages of aging is essential to prevent and accomplish with effective interventions to delay the onset of major oral function deterioration, and ultimately extend the healthy life expectancy of the elderly.

Moreover, this study also used factor analysis to reduce the number of variables into a smaller set of factors. Therefore, this study's findings showed that the occurrence of OHF, based on a set of oral function parameters, is increased with age, as indicated by two major domains of OHF—"chewing-related" and "tongue-related" factors were identified as influenced by age. However, the "oral status" domain was not associated with higher risk if OHF with advancing age in this sample of healthy subjects. Nevertheless, oral status associated with poor oral cleanliness and hyposalivation may be associated with older ages and frail subjects, and results may vary from this study with healthy volunteers and, therefore, further studies with older subjects specifically designed to address those issues should be considered.

## 5. Conclusions

The findings suggest that advancing age is associated with deterioration of oral function, particularly in chewing and tongue-related activities, contributing to increased OHF in older adults. The results of this exploratory study corroborate with the importance of early detection and targeted interventions to maintain oral health and function in the aging population, with a focus on preserving chewing and tongue-related capabilities.

## Figures and Tables

**Table 1:** Summary data of the participants according to the age groups.

	Age categories						Total	*p* value
	20–29 years	30–39 years	40–49 years	50–59 years	60–69 years	≥70 years		
BMI ≥ 25	6 (28.6)	9 (45.0)	9 (45.0)	16 (80.0)	16 (81.8)	11 (47.8)	69 (54.8)	0.010^a^
Handgrip force	36.5 (1.8)	32.7 (14.1)	30.2 (13.5)	28.4 (10.5)	25.8 (7.8)	20.8 (9.5)	28.8 (12.1)	<0.001^b^
Use of denture	0 (0.0)	2 (10.0)	4 (20.0)	10 (50.0)	11 (50.0)	13 (56.5)	40 (31.7)	<0.001^a^
Number of teeth	28.9 (2.1)	27.7 (3.7)	24.7 (5.2)	20.1 (7.5)	16.6 (9.3)	17.7 (7.4)	22.4 (7.9)	<0.001^b^
Root stumps present	0 (0.0)	1 (5.0)	1 (5.0)	1 (5.0)	2 (9.1)	2 (8.7)	7 (5.6)	0.466^a^
Dental implants present	0 (0.0)	0 (0.0)	1 (5.0)	3 (15.0)	5 (22.7)	2 (8.7)	11 (8.7)	0.145^a^
Total	21	20	20	20	22	23	126	—

*Note:* Data are expressed as means (standard deviations) for numerical data, and frequencies (percent) for nominal data.

^a^Chi-square for trend.

^b^One-way ANOVA.

**Table 2:** Summary data of the oral function parameters according to the age groups.

Oral function parameter		Age categories						Total	*p* value⁣^*∗*^	Effect size⁣^*∗∗*^
		20–29 years	30–39 years	40–49 years	50–59 years	60–69 years	≥70 years			
Oral hygiene (CFU/mL)		4.08 (1.36)	3.97 (1.47)	3.97 (1.29)	3.12 (1.41)	3.30 (1.44)	3.84 (1.13)	3.71 (1.37)	0.071	0.081
Oral dryness		28.9 (1.4)	29.0 (2.0)	28.6 (1.8)	28.2 (1.9)	26.6 (5.4)	27.7 (1.2)	28.1 (2.9)	0.064	0.083
Occlusal force (N)		854 (298)^a^	905 (510)^a^	578 (357)^ab^	587 (460)^ab^	409 (240)^b^	460 (380)^b^	629 (420)	<0.001	0.204
Oral diadochokinesis		6.70 (0.84)^a^	6.04 (1.04)^ab^	5.90 (0.87)^ab^	5.36 (1.27)^bc^	5.22 (1.19)^bc^	4.80 (0.96)^c^	5.65 (1.20)	<0.001	0.279
Tongue pressure (kPa)		36.9 (10.2)^a^	34.9 (11.2)^a^	32.8 (10.1)^ab^	32.6 (10.1)^ab^	28.3 (13.1)^ab^	23.0 (11.0)^b^	31.2 (11.8)	0.002	0.152
Masticatory function (mg/dL)		220.9 (74.0)^a^	176.1 (49.2)^ab^	150.7 (60.3)^bc^	115.6 (65.2)^c^	119.5 (54.3)^bc^	115.8 (74.1)^c^	149.5 (74.1)	<0.001	0.280
Swallowing function (EAT10)		0.238 (0.70)	0.050 (0.22)	0.700 (1.75)	0.500 (1.27)	1.00 (2.27)	1.87 (3.47)	0.754 (2.04)	0.141	0.066
Number of failed oral function tests	0 – 2	19 (90.5)	14 (70.0)	12 (60.0)	8 (40.0)	8 (36.4)	2 (8.7)	63 (50.0)	<0.001	0.315
	3 – 4	2 (9.5)	5 (25.0)	7 (35.0)	10 (50.0)	8 (36.4)	14 (60.9)	46 (36.5)	—	—
	5 – 7	0 (0.0)	1 (5.0)	1 (5.0)	2 (10.0)	6 (27.3)	7 (30.4)	17 (13.5)	—	—

*Note:* Data are expressed as means (and standard deviations). Different letters indicate different groups in pairwise comparisons.

Abbreviation: ES, effect size.

⁣^*∗*^Kruskall-Walis test.

⁣^*∗∗*^ES—small effect = 0.01–<0.06; moderate effect = 0.06–<0.14; large effect = ≥0.14.

**Table 3:** Total variance explained for the three extracted components.

Component	Initial eigenvalues			Rotation sums of squared loadings		
	Total	Variance (%)	Cumulative (%)	Total	Variance (%)	Cumulative (%)
1	1548	22.117	22.117	1435	20.497	20.497
2	1304	18.630	40.747	1309	18.703	39.200
3	1111	15.872	56.619	1219	17.419	56.619

*Note:* Extraction method: principal component analysis (PCA).

**Table 4:** Rotated component matrix: factor loadings after varimax rotation.

Domains	Parameters	Component^a^		
		1	2	3
Chewing-related	Occlusal force (*N*)	0.766	—	—
	Masticatory function (mg/dL)	0.731	—	—

Tongue-related	Tongue pressure (kPa)	—	0.807	—
	Oral diadochokinesis	—	0.593	—
	Swallowing function (EAT10)	—	0.548	—

Oral status	Oral dryness	—	—	0.772
	Oral hygiene (CFU/mL)	—	—	0.555

^a^The strongest associations between questions and domains are presented.

## Data Availability

Data will be made available upon a written request to the corresponding author.
